# Therapeutic Effect of *Scutellaria baicalensis* on L-Thyroxine-Induced Hyperthyroidism Rats

**DOI:** 10.1155/2019/3239649

**Published:** 2019-09-15

**Authors:** Mia Kim, Byung-Cheol Lee

**Affiliations:** ^1^Department of Cardiovascular and Neurologic Disease (Stroke Center), College of Korean Medicine, Kyung Hee University, 23 Kyungheedae-ro, Dongdaemun, Seoul 02447, Republic of Korea; ^2^Department of Clinical Korean Medicine, Graduate School, Kyung Hee University, 26 Kyungheedae-ro, Dongdaemun-gu, Seoul 02447, Republic of Korea

## Abstract

**Background:**

This study was performed to evaluate the anti-hyperthyroidal effects and action mechanism of *Scutellaria baicalensis* Georgi (SB), a medicinal herb, on levothyroxine (LT4)-induced hyperthyroidal rats.

**Methods:**

Male Wistar rats were divided into five groups, namely, euthyroidal normal group (Normal), hyperthyroidism control group (Control), hyperthyroidism plus PTU-treated group (PTU) as a positive control, hyperthyroidism plus 400 mg/kg SB-treated group (SB400), and hyperthyroidism plus 800 mg/kg SB-treated group (SB800). The rats in groups other than Normal were injected with LT4 for 2 weeks to induce hyperthyroidism and then were administrated each treatment for 2 weeks. Clinical symptoms and biomarkers related to hyperthyroidism were examined, and the gene expressions related to the regulation of thyroid hormone were determined.

**Results:**

Compared with the Control group, pulse rate, serum T3, T4, triglyceride, thyroid follicle size, and the deiodinase 1 (Dio1) gene expression were significantly reduced in the SB and PTU groups. Serum TSH and the thyroxine-binding globulin (Tbg) gene expression were significantly increased in the SB and PTU groups.

**Conclusions:**

These results suggest that SB might suppress T3, T4, and adrenergic activity by modulating Dio1 and Tbg expression, and therefore, SB could be an alternative therapy for hyperthyroidism.

## 1. Introduction

Hyperthyroidism is a hypermetabolic condition of thyrotoxicosis resulting from an overproduction of thyroid hormone in the thyroid gland [1]. Graves' disease, the most common cause of hyperthyroidism, manifests the clinical symptoms of goiter, palpitation, sweating, weight loss, and ophthalmopathy and laboratory findings of increased levels of T3, T4, and TSH receptor antibody and decreased TSH [2]. The cause of Graves' disease is that when MHC class II is demonstrated in the epithelial cell of the thyroid, T cells recognize the thyroid-stimulating hormone receptor (TSHR) as an extrinsic antigen and create autoantibodies from the B cell [3]. This stimulates the thyroid receptor resulting in a hypersynthesis and secretion of the thyroid hormone [4]. Thus, for a complete fundamental treatment, the production of autoantibody which stimulates TSH receptors should be suppressed, but this is unavailable in current clinical trials [5]. Instead, methods of destroying or removing thyroid tissue by radioactive iodine or surgery, or using a drug that restrains the production and distribution of thyroid hormones are used to maintain normal thyroid function [6]. However, it is difficult to expect a clinically complete remission of Graves' disease with such treatments [5]. Frequent relapses and side effects often follow drug treatments, while radioactive iodine therapy and thyroidectomy may lead to hypothyroidism [7]. Especially in countries like Korea and Japan with high reliance upon antithyroid drugs, where the proportion of drug treatment accounted for 80 to 88 percent of the treatments, development of a treatment to supplement or replace the use of antithyroid drugs is desperately needed [8]. Today, due to such problems of the commonly used treatments, resistance to antithyroid drugs, and the tedious clinical improvement of these treatments, more patients are willing to cure their disease through herbal remedies in clinical practice [9]. Ahnjeonbaekho-tang (AJBHT) has been used as an herbal remedy for Graves' disease [10]. Among the components of AJBHT, daidzein and baicalein are known to have antithyroid effects [11]. Clinical studies showed that AJBHT reduced the level of thyroid hormone in Graves' disease patients who were resistant to methimazole (MMI) [12]. In in vitro study, AJBHT was suggested to have a different mechanism of modulating cyclic AMP and Tbg expression [13]. In contrast, MMI reduced the synthesis of thyroid hormone by inhibiting thyroid peroxidase (TPO) activity [14]. In this study, we evaluate the anti-hyperthyroidal effects and action mechanism of the *Scutellaria baicalensis* Georgi (SB), a main herb of AJBHT, on levothyroxine (LT4)-induced hyperthyroidal rats.

## 2. Materials and Methods

### 2.1. Preparation of SB

SB was purchased from the Department of Pharmaceutical Preparation of the Hospital of Korean Medicine, Kyung Hee University (Seoul, Korea). One thousand grams of SB was boiled with 1,500 mL of 80% ethanol using a heating mantle for 2 hours. The extract was transferred to a 500 mL flask by an applicator and filtered. The filtrate was concentrated with a rotary evaporator (Model NE-1, EYELA Co., Japan). The extract was freeze-dried and stored at room temperature. The final extraction yield of SB was 33%.

### 2.2. Animal Model and Treatment

Six-week-old male Wistar rats were purchased from the Central Lab. Animal Inc. (Seoul, Korea). They were in a moisture-controlled room (40–70%) with a 12-hour light-dark cycle and allowed access to water and diet *ad libitum*. After 1-week period of acclimation, every rat except the Normal group was daily subcutaneously injected with levothyroxine (LT4) (Sigma, MO, USA) at a dose of 0.3 mg/kg for 2 weeks for inducing hyperthyroidism. The rats were divided into five groups: euthyroidal normal group (Normal, *n*=6), LT4-injected hyperthyroidal control group (Control, *n*=6), LT4 plus 10 mg/kg propylthiouracil-treated group (Sigma, MO, USA) (PTU, *n*=6) as a positive control, LT4 plus 400 mg/kg SB-treated group (SB400, *n*=6), and LT4 plus 800 mg/kg SB-treated group (SB800, *n*=6). The dosages of SB extracts and PTU used in this study were selected based on the previous report. SB extract was orally administered once a day for 14 days from 15th LT4 treatment, and PTU was intraperitoneally injected. The body weight of each rat was measured at the beginning and before the final sampling. The total amount of food consumption was recorded every day. To assess the food intake, the total consumption of food during a day was measured in every cage. Then the 1-day consumption of each rat was calculated by dividing into the number of the rats in each cage. At week 4, the rats were sacrificed and the weights of thyroid and livers were measured. This study was approved by the Institutional Animal Care and Use Committee of Kyung Hee University, Seoul, Korea.

### 2.3. Measurement of Pulse Rate and Oxygen Saturation

The pulse rate and oxygen saturation of rats were measured using a stand-alone pulse oximeter (Med Associates Inc., VT). Each rat was placed at the center of the fixing apparatus (Kenis, Japan) and was kept for 5 minutes until motionless, and then the wrap sensor was securely held in the tail. Once a stable signal was acquired, the data were recorded using the pulse oximeter software (Med Associates Inc., VT) for analysis.

### 2.4. Measurement of Serum Thyroid Hormones

Blood samples were collected, and serum was separated by centrifugation at 3000 rpm for 10 min at 4°C. Serum levels of triiodothyronine (T3), T4, and thyroid-stimulating hormone (TSH) were analyzed by colorimetric competitive enzyme immunoassay using individual ELISA Kit (Cusabio, China). In detail, microtiter wells coated with antibody were prepared and 100 *μ*l of samples and standard T3, T4, or TSH solution were applied, then followed by 50 *μ*l of HRP conjugate, 50 *μ*l of color solution, and 50 *μ*l of stop solution. The absorbance was measured by an ELISA reader at 450 nm.

### 2.5. Biochemical Assays

At the end of the experiment, after 14-hour fasting, serum total cholesterol (TC), high-density lipoprotein (HDL) cholesterol, low-density lipoprotein (LDL) cholesterol, triglyceride (TG), aspartate transaminase (AST), alanine aminotransferase (ALT), and creatinine levels were measured.

### 2.6. RNA Extraction and Analysis of Gene Expression

At week 4, the rats were sacrificed and the livers were dissected. RNA extraction was performed using a Mini RNA Isolation IITM (Zymo Research Corp, CA, USA). RNA was extracted using TRIzol reagent. To evaluate gene expression including deiodinase 1 (Dio1), thyroid hormone responsive spot 14 (Thrsp or Spot14), and thyroxine-binding globulin (Tbg), quantitative real-time polymerase chain reaction (qRT-PCR) was performed. Prior to qRT-PCR, the complementary DNA (cDNA) was synthesized using an Advantage RT for PCR Kit (Clontech, USA). To the cDNA obtained through reverse transcription PCR, 2x SYBR reaction buffer, primers, and dH_2_O were added, and qRT-PCR was carried out using 7900HT Fast Real-Time PCR System (Applied Biosystems®, USA). The primer sequencing is as follows: *Dio1*, 5′-TTTAAGAACAACGTGGACATCAGG-3′ and 5′-GGTTTACCCTTGTAGCAGATCCT-3′; *Spot14*, 5′-CTTACCCACCTGACCCAGAA-3′ and 5′-CATCGTCTTCCCTCTCGTGT-3′; *Tbg*, 5′-GCTGCTTTAGCCATGCTTTC-3′ and 5′-AAACTGCATTTCCCATCTGC-3′; and *GAPDH,* 5′-GTCGGTGTCAACGGATTTG-3′ and 5′-AGCTTCCCATTCTCAGCC-3′. For gene expression analysis, the threshold cycle for each gene, obtained with SDS Software 2.4 (Applied Biosystems®, USA), was converted to relative quantitation based on GAPDH, and the fold change was calculated. The fold change value of each experimental group was normalized according to the Normal group, which was defined as 1.

### 2.7. Histomorphometric Analyses of Thyroid

Obtained thyroid samples were fixed in 10% neutral buffered formalin and embedded in paraffin to make paraffin blocks. Each block was sliced into 4 *μ*m thick sections with a microtome and attached to a gelatin-coated slide. Two sections per animal were stained with hematoxylin and eosin, and digital images were obtained using a high-resolution camera-mounted optical microscope (Olympus BX-50, Olympus Optical, Tokyo, Japan) connected to a computer. Using ImageJ, the thyroid follicular lumen area in thyroid tissue was measured.

### 2.8. Statistical Analysis

Statistical analyses were performed using GraphPad Prism 6 (GraphPad Software Inc., San Diego, USA). Statistical comparisons between the groups were performed with one-way analysis of variance (ANOVA), followed by Tukey's post hoc test. The data are presented as mean ± SEM. A two-tailed *P* value of <0.05 was considered statistically significant.

## 3. Results

### 3.1. Effects of SB on Body Weight and Thyroid and Liver Weight in Hyperthyroidal Rats

**Figure 1:**
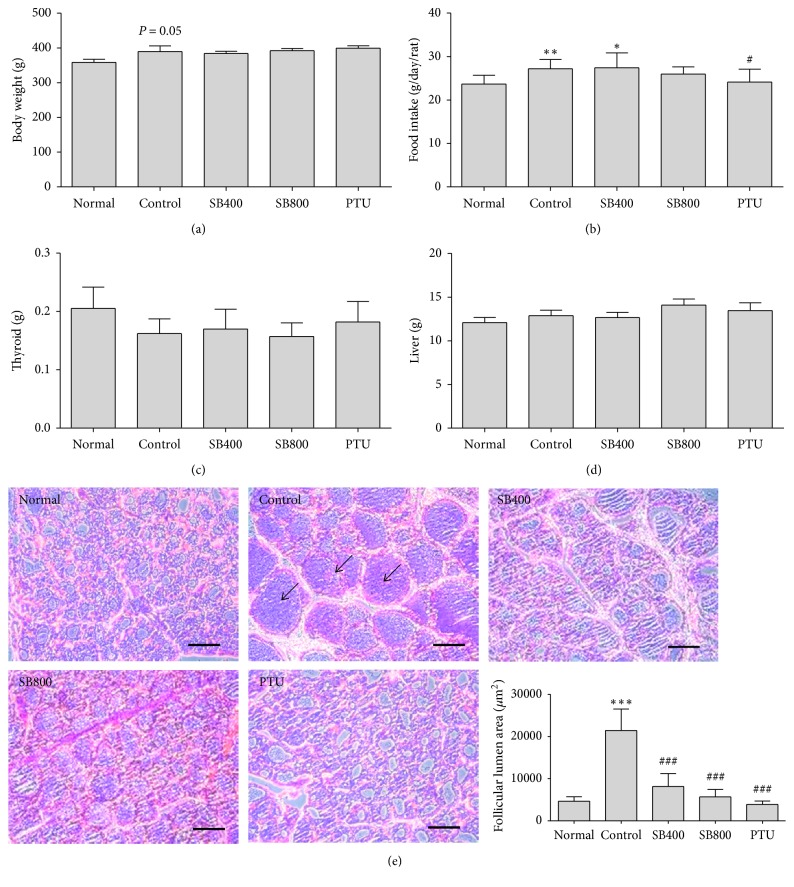
The changes by SB on body weight (a), food intake (b), thyroid weight (c), liver weight (d), and histological changes in thyroid (e). Representative histological images were assessed by hematoxylin and eosin (H&E) staining, scale bar indicates 100 *μ*m, and arrow indicates thyroid follicular lumen (e). *N*=6 in each group. Data shown as mean ± standard error of the mean (SEM). ^*∗*^*P* < 0.05, ^*∗∗*^*P* < 0.01, ^*∗∗∗*^*P* < 0.001 versus Normal group; ^#^*P* < 0.05, ^###^*P* < 0.001 versus Control group.

The body weight of the hyperthyroidal control group increased to 389.57 ± 9.19 g, while that of the Normal group was 358.5 ± 9.18 g (*P*=0.05). However, the body weight of the SB and PTU groups was no different compared to the Control group (Figure 1(a)). The amount of food intake was also examined to determine if the weight loss was due to a decrease in intake. Compared with the Normal group, there was a significant increase in food intake in the hyperthyroidal control group (*P* < 0.01) and SB400 group (*P* < 0.05) but not in the SB800 and PTU groups (Figure 1(b)). The weight of thyroid and liver in the hyperthyroidal control group did not show the differences compared to the Normal group and SB and PTU groups (Figures 1(c) and 1(d)).

### 3.2. Effects of SB on Thyroid Hormones in Hyperthyroidal Rats

**Figure 2:**
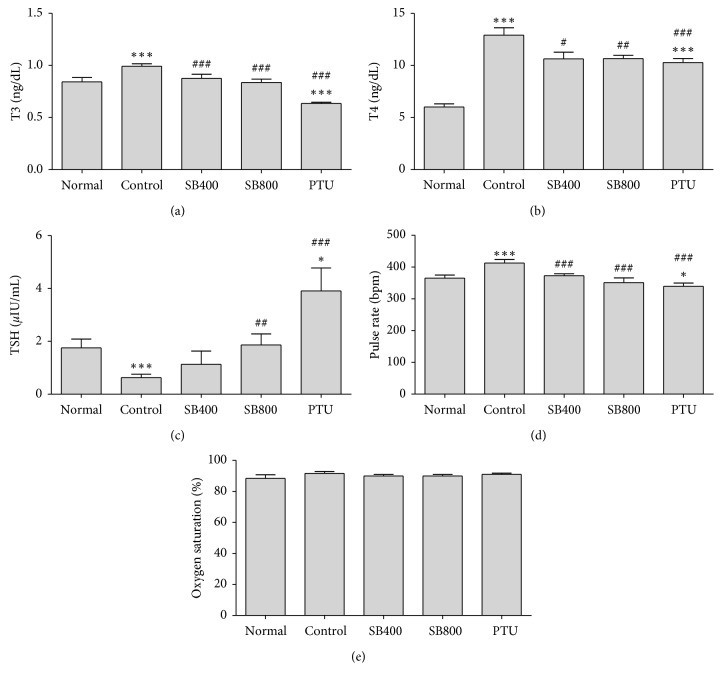
The changes by SB on of triiodothyronine (T3) (a), thyroxine (T4) (b), thyroid-stimulating hormone (TSH) (c), pulse rate (d), and oxygen saturation (e). Blood samples were obtained at week 4. *N*=6 in each group. Data shown as mean ± SEM. ^*∗*^*P* < 0.05, ^*∗∗*^*P* < 0.01, ^*∗∗∗*^*P* < 0.001 versus Normal group; ^#^*P* < 0.05, ^##^*P* < 0.01, ^###^*P* < 0.001 versus Control group.

As expectation, serum T4 levels in the hyperthyroidal control group (12.90 ± 0.71 ng/ml) were significantly elevated compared to the Normal group (6.01 ± 0.29 ng/ml, *P* < 0.001). SB significantly reduced T4 level in the SB400 group (10.62 ± 0.64 ng/ml, *P* < 0.05) and in the SB800 group (0.66 ± 0.31 ng/ml, *P* < 0.01) compared to the Control group, which showed similar effect to the PTU group (10.27 ± 0.40 ng/ml, *P* < 0.001) (Figure 2(b)). As a consequence of T4 elevation, serum T3 levels in the hyperthyroidal control group (0.99 ± 0.02 ng/ml) were also significantly elevated compared to the Normal group (0.84 ± 0.04 ng/ml, *P* < 0.01). SB significantly reduced T3 level in both the SB400 group (0.87 ± 0.04 ng/ml, *P* < 0.05) and SB800 group (0.83 ± 0.03 ng/ml, *P* < 0.01) compared to the Control group, and the PTU group showed a great reduction in T3 level (0.63 ± 0.01 ng/ml, *P* < 0.001) (Figure 2(a)). Serum TSH levels in the hyperthyroidal control group (0.62 ± 0.13 ng/ml) were significantly decreased compared to the Normal group (1.75 ± 0.34 *μ*IU/ml, *P* < 0.001). SB significantly improved TSH level in the SB800 group (1.86 ± 0.41 *μ*IU/ml, *P* < 0.01) compared to the Control group, and TSH level in the PTU group (3.91 ± 0.87 *μ*IU/ml) increased higher than that of the Control and Normal groups (*P* < 0.001, *P* < 0.001, respectively) (Figure 2(c)).

### 3.3. Effects of SB on Pulse Rate and Oxygen Saturation in Hyperthyroidal Rats

The injection of LT4 significantly raised pulse rate in the hyperthyroidal control group (412.69 ± 10.84 bpm) compared to the Normal group (365.21 ± 9.54 bpm, *P* < 0.001), and SB400 (372.52 ± 6.36, *P* < 0.01), SB800 (350.88 ± 14.78, *P* < 0.01), and PTU (339.29 ± 10.25 bpm, *P* < 0.001) treatment decreased (Figure 2(d)). The oxygen saturation was slightly increased in the LT4-injected hyperthyroidal control group without significance (Figure 2(e)).

### 3.4. Effects of SB on Lipid Profile and Other Biochemical Profiles in Hyperthyroidal Rats

**Figure 3:**
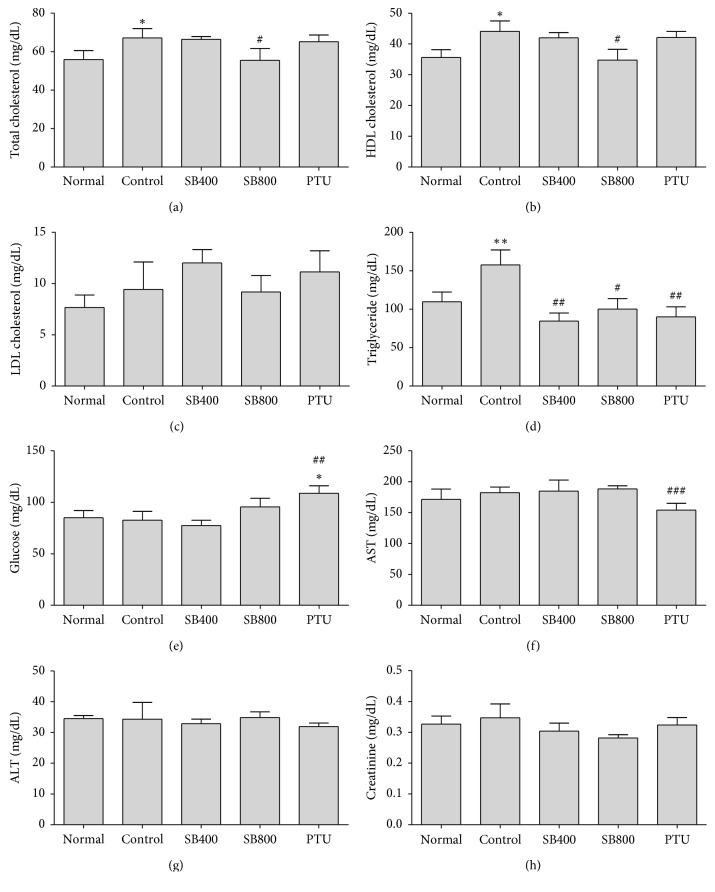
Effects of SB on total cholesterol (a), HDL cholesterol (b), LDL cholesterol (c), triglyceride (d), glucose (e), aspartate transaminase (AST) (f), alanine aminotransferase (ALT) (g), and creatinine (h). Blood samples were obtained at week 4. *N*=6 in each group. Data shown as mean ± SEM. ^*∗*^*P* < 0.05, ^*∗∗*^*P* < 0.01 versus Normal group; ^#^*P* < 0.05, ^##^*P* < 0.01, ^###^*P* < 0.001 versus Control group.

The injection of LT4 increased food intake and body weight, which induced increase in total cholesterol (Figure 3(a)), HDL cholesterol (Figure 3(b)), and triglyceride (Figure 3(d)) in the hyperthyroidal control group compared to the Normal group (*P* < 0.05, *P* < 0.05, *P* < 0.05, respectively), and SB400 decreased TG (*P* < 0.05), SB800 decreased total cholesterol (*P* < 0.05), HDL cholesterol (*P* < 0.05), and TG (*P* < 0.05), and PTU decreased TG (*P* < 0.01). The serum glucose (Figure 3(e)), AST (Figure 3(f)), and ALT (Figure 3(g)) did not show differences among the Normal, hyperthyroidal control, and SB-treated groups, but PTU significantly increased glucose compared to the Control (*P* < 0.01) and Normal groups (*P* < 0.05) and decreased AST compared to the Control group (*P* < 0.05). There was no difference among all groups in creatinine (Figure 3(h)).

### 3.5. Effects of SB on the Thyroid Follicular Lumen Area in Hyperthyroidal Rats

Because LT4 injection which suppresses pituitary TSH synthesis induces the decrease of thyroid cell proliferation [15], we analyzed the thyroid follicular lumen area followed by H&E stain. While marked increases in colloid space due to atrophic changes of follicular cells were observed in the hyperthyroidal control group, SB and PTU treatment restored it (Figure 1(d)). In the histomorphometric analysis, the thyroid follicular lumen area in the hyperthyroidal control group (21429.65 ± 5098.02 *μ*m^2^) was significantly increased compared to the Normal group (4648.60 ± 1077.72 *μ*m^2^, *P* < 0.001). SB significantly reduced the thyroid follicular lumen area in the SB400 group (8185.35 ± 3049.92 *μ*m^2^, *P* < 0.001) and in the SB800 group (5691.67 ± 1764.79 *μ*m^2^, *P* < 0.001) compared to the Control group, and PTU group showed a great decrease in the thyroid follicular lumen area (3901.213 ± 822.24 *μ*m^2^, *P* < 0.001) (Figure 1(d)).

### 3.6. Effects of SB on Thyroid Hormone-Regulated Gene Expression of Liver in Hyperthyroidal Rats

The LT4 injection changes the expression of thyroid hormone-regulated genes in the liver, so we analyzed the thyroid hormone-regulated gene expression including *Dio1*, *Spot14*, and *Tbg* in liver tissue.

**Figure 4:**
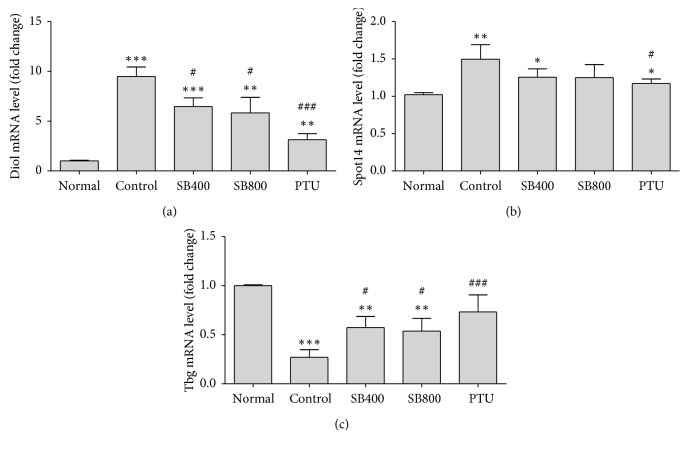
The changes by SB on thyroid hormone-regulated genes including deiodinase 1 (Dio1) (a), thyroid hormone responsive spot 14 (Thrsp or Spot14) (b), and thyroxine-binding globulin (Tbg) (c) expression in liver tissue. Liver tissue was obtained at week 4, and quantitative RT-PCR was used to measure. Gene expression was normalized to that of GAPDH. *N*=6 in each group. Data shown as mean ± SEM. ^*∗*^*P* < 0.05, ^*∗∗*^*P* < 0.01, ^*∗∗∗*^*P* < 0.001 versus Normal group; ^#^*P* < 0.05, ^##^*P* < 0.01, ^###^*P* < 0.001 versus Control group.

The *Dio1* and *Spot14* expression levels were significantly increased in the hyperthyroidal control group relative to the Normal group (*Dio1,P* < 0.001; *Spot14,P* < 0.01), and SB significantly decrease the *Dio1* levels in both SB400 (*P* < 0.05) and SB800 groups (*P* < 0.05), and PTU also decreased level (*P* < 0.001) compared to the Control group (Figure 4(a)), but in case of *Spot14* expression, only PTU significantly decreased the level (*P* < 0.05) (Figure 4(b)). The expression level of *Tbg* in the hyperthyroidal control group was significantly downregulated compared to the Normal group (*P* < 0.001). SB significantly upregulated the expression level in the SB400 group (*P* < 0.05) and in the SB800 group (*P* < 0.05) compared to the Control group, and PTU group showed great upregulation of *Tbg* level (*P* < 0.01) (Figure 4(c)).

## 4. Discussion

Hyperthyroidism is characterized by palpitation, weight loss, increased appetite, and anxiety, which is similar to a state of increased adrenergic activity [16]. In thyrotoxicosis, plasma catecholamines are unchanged, and the beta-adrenergic receptor density is altered in a time- and tissue-dependent manner, resulting in increased tissue sensitivity to catecholamines [17]. In this study, we directly injected levothyroxine (LT4) at a dose of 0.3 mg/kg to rats for 2 weeks, causing thyrotoxic state, and we examined body weight, appetite, heart rate, and the level of thyroid hormone. Food intake significantly increased in the hyperthyroidal control group and SB400 group but not in the SB800 and PTU groups. Body weight actually increased in the hyperthyroidal control group. This result seems to be due to the short duration of hypermetabolic state, along with the increase in food intake of the rats in the Control group. In terms of the thyroid hormone, SB significantly reduced T3 and T4 levels in the SB400 group and in the SB800 group compared to the Control group, which showed similar effects to the PTU. With decreased T3 and T4, the TSH level increased in the SB800 and PTU groups. The Control group in a thyrotoxic state showed increased heart rate, but the heart rate decreased in the SB400, SB800, and PTU groups. The heart relies mainly on the action of T3, since T3 is transported into the myocyte [18]. Most of T4, acting mostly as a prohormone, is converted to biologically active T3 through the removal of iodide by deiodinases [19]. Type 1 deiodinase (Dio1) activates the thyroid hormone by converting T4 to active T3, and it deactivates the thyroid hormone by converting T4 to inactive reverse T3 (rT3) or to T2 [20]. Both T4 and T3 circulate in the blood almost entirely bound to thyroxine-binding globulin [21]. The remaining unbound T3 is transported through a variety of membrane transport proteins and subsequently to the cell nucleus to regulate the expression of selected genes. Therefore, deiodinases are critical for biological effects mediated by thyroid hormone [22]. In this study, we found decreased expression of *Dio1* and upregulated level of Tbg in the SB400, SB800, and PTU groups. In contrast, hyperthyroidal control group revealed increased expression level of *Dio1* and downregulated level of Tbg. SB and PTU suppressed the action of Dio1, therefore inhibiting the conversion of T4 into an active unbounded T3. These results suggest that SB might suppress T3, T4, and adrenergic activity by modulating Dio1 and Tbg expression. In Asia, SB has been widely used for treating cardiovascular disease and anxiety disorder [23], and SB seems to be helpful for treating these diseases by regulating thyroid hormone.

## 5. Conclusions

Based on these results, we conclude that SB improved thyroid hormones, adrenergic activity, and lipid metabolism in LT4-induced hyperthyrodism rats. Our findings suggest that these antithyroidal, antiadrenergic, and antilipid effects of SB could be mediated by the modulation of thyroid hormone-regulated gene expression. For the clinical use of SB, further clinical research and mechanism study on other related factors is needed.

## Data Availability

The data used to support the findings of this study are available from the corresponding author upon request.

## References

[1] De Leo S., Lee S. Y., Braverman L. E. (2016). Hyperthyroidism. *The Lancet*.

[2] Ginsberg J. (2003). Diagnosis and management of Graves’ disease. *CMAJ*.

[3] Smith T. J., Hegedus L. (2016). Graves’ disease. *New England Journal of Medicine*.

[4] Antonelli A., Ferrari S. M., Corrado A., Di Domenicantonio A., Fallahi P. (2015). Autoimmune thyroid disorders. *Autoimmunity Review*.

[5] Bahn R. S. (2012). Autoimmunity and Graves’ disease. *Clinical Pharmacology and Therapeutics*.

[6] Klein I., Becker D. V., Levey G. S. (1994). Treatment of hyperthyroid disease. *Annals of Internal Medicine*.

[7] Cooper D. S. (2005). Antithyroid drugs. *New England Journal of Medicine*.

[8] Wartofsky L., Glinoer D., Solomon B. (1991). Differences and similarities in the diagnosis and treatment of Graves’ disease in Europe, Japan, and the United States. *Thyroid*.

[9] Zen X. X., Yuan Y., Liu Y., Wu T. X., Han S. (2007). Chinese herbal medicines for hyperthyroidism. *Cochrane Database System Reviews*.

[10] Soon-Il K., Ki-Hoon K., Young-Seok K. (2005). The clinical effects of Ahnjeonbaekho-tang (AJBHT) on Graves’ disease. *The Journal of Korean Medicine*.

[11] Ferreira A. C., Lisboa P. C., Oliveira K. J., Lima L. P., Barros I. A., Carvalho D. P. (2002). Inhibition of thyroid type 1 deiodinase activity by flavonoids. *Food and Chemical Toxicology*.

[12] Li H., Okuda J., Akamizu T., Mori T. (1995). A hyperthyroid patient with Graves’ disease who was strongly resistant to methimazole: investigation on possible mechanisms of the resistance. *Endocrine Journal*.

[13] Lee B. C., Kang S. I., Ahn Y. M., Doo H. K., Ahn S. Y. (2008). An alternative therapy for Graves’ disease: clinical effects and mechanisms of an herbal remedy. *Biological & Pharmaceutical Bulletin*.

[14] Roy G., Mugesh G. (2005). Anti-thyroid drugs and thyroid hormone synthesis:  effect of methimazole derivatives on peroxidase-catalyzed reactions. *Journal of the American Chemical Society*.

[15] Hood A., Liu Y. P., Gattone V. H., Klaassen C. D. (1999). Sensitivity of thyroid gland growth to thyroid stimulating hormone (TSH) in rats treated with antithyroid drugs. *Toxicol Sciences*.

[16] Ertek S., Cicero A. F. (2013). Hyperthyroidism and cardiovascular complications: a narrative review on the basis of pathophysiology. *Archives of Medical Science*.

[17] Vargas-Uricoechea H., Bonelo-Perdomo A., Sierra-Torres C. H. (2014). Effects of thyroid hormones on the heart. *Clínica e Investigación en Arteriosclerosis*.

[18] Klein I., Danzi S. (2007). Thyroid disease and the heart. *Circulation*.

[19] Danzi S., Klein I. (2014). Thyroid disease and the cardiovascular system. *Endocrinology and Metabolism Clinics of North America*.

[20] Bianco A. C., Salvatore D., Gereben B., Berry M. J., Larsen P. R. (2002). Biochemistry, cellular and molecular biology, and physiological roles of the iodothyronine selenodeiodinases. *Endocrine Reviews*.

[21] Schussler G. C. (2002). The thyroxine-binding proteins. *Thyroid*.

[22] Brent G. A. (2012). Mechanisms of thyroid hormone action. *Journal of Clinical Investigation*.

[23] Wang C.-Z., Mehendale S. R., Calway T., Yuan C.-S. (2011). Botanical flavonoids on coronary heart disease. *The American Journal of Chinese Medicine*.

